# Traditional and Latest Researches on *Aspergillus oryzae* and Related *Koji* Molds

**DOI:** 10.3390/jof7121075

**Published:** 2021-12-14

**Authors:** Yujiro Higuchi, Katsuhiko Kitamoto

**Affiliations:** 1 Department of Bioscience and Biotechnology, Faculty of Agriculture, Kyushu University, 744 Motooka, Fukuoka 819-0395, Japan; 2 Department of Pharmaceutical and Medical Business Sciences, Nihon Pharmaceutical University, Yushima, Bunkyo-ku, Tokyo 113-0034, Japan; k-kitamoto@nichiyaku.ac.jp

We would like to thank all the contributors to this Special Issue on *Aspergillus oryzae* and related *Koji* molds (https://www.mdpi.com/journal/jof/special_issues/Aspergillus_Koji, accessed on 9 December 2021). Collectively, in this Special Issue, nine reviews and one article have been published to summarize traditional and latest researches about the yellow *Koji* molds *Aspergillus oryzae* and *Aspergillus sojae*, the black *Koji* mold *Aspergillus luchuensis*, and the white *Koji* mold *Aspergillus luchuensis* mut. *kawachii* (Figure 1).

Spores of *Koji* molds are called *Koji* starter, and these cultures on cereals and crops are used for fermented foods in industries. Yamashita has outlined the historical backgrounds of *Koji* starter and *Koji*, explained these varieties for preparations of fermented foods, and proposed potential uses in the future [1]. One of the fermented beverages produced by using *A. oryzae* is *amazake*, the Japanese traditional sweet drink. Kurahashi has provided the information from recent research about ingredients and functionality of *amazake* [2]. In addition, Kurahashi et al. have reported the latest research data about the improvement of defecation frequency by taking *Koji amazake* [3]. *Shochu* and *awamori*, the Japanese traditional distilled liquors, are made by using *A. kawachii* and *A. luchuensis*, respectively. Hayashi et al. have described the preparation methods of *shochu* and *awamori* with molecular backgrounds and diverse flavor [4]. *Miso*, a traditional Japanese seasoning paste, is made of soybeans and rice fermented with *A. oryzae*. Varieties of *miso*, their preparations with responsible enzymes, and their nutritional components and functions have been covered by Kusumoto et al. [5]. Ito and Matsuyama have detailed the making processes of the Japanese soy sauce, in which *Koji* molds are essential for producing varieties of crucial enzymes [6]. They have also summarized how glutamate (umami) is generated in soy sauce making. *Koji* molds are very useful, not only for producing fermented foods and beverages, but also for supplying valuable materials. Kitagaki has listed substances for medical applications that are produced by *Koji* molds [7]. Due to a great ability in the production of valuable material, including enzyme secretion, the molecular mechanisms of secretory pathways in *A. oryzae* have been well investigated. Higuchi has covered such recent advances on cell biological aspects of membrane traffic in *Koji* molds [8]. Not only proteins but also secondary metabolites (SMs) are abundantly produced in *Koji* molds. Awakawa and Abe have introduced how *A. oryzae* is a useful heterologous host for producing SMs, such as polyketide-derived meroterpenoids in a group of bioactive natural products [9]. Lastly, Maruyama has covered the genome editing technology CRISPR/Cas9 system in *A. oryzae*, which has been a powerful molecular tool to develop industrial *Koji* mold strains [10]. All papers in this Special Issue have provided the traditional and increasing knowledge of *A. oryzae* and related *Koji* molds with insightful perspectives that will direct future research in each field of study.

Again, we wish to express thanks to all authors and reviewers for their pivotal contributions to this Special Issue, which has been made a highly successful and timely collection of studies.

## Figures and Tables

**Figure 1:**
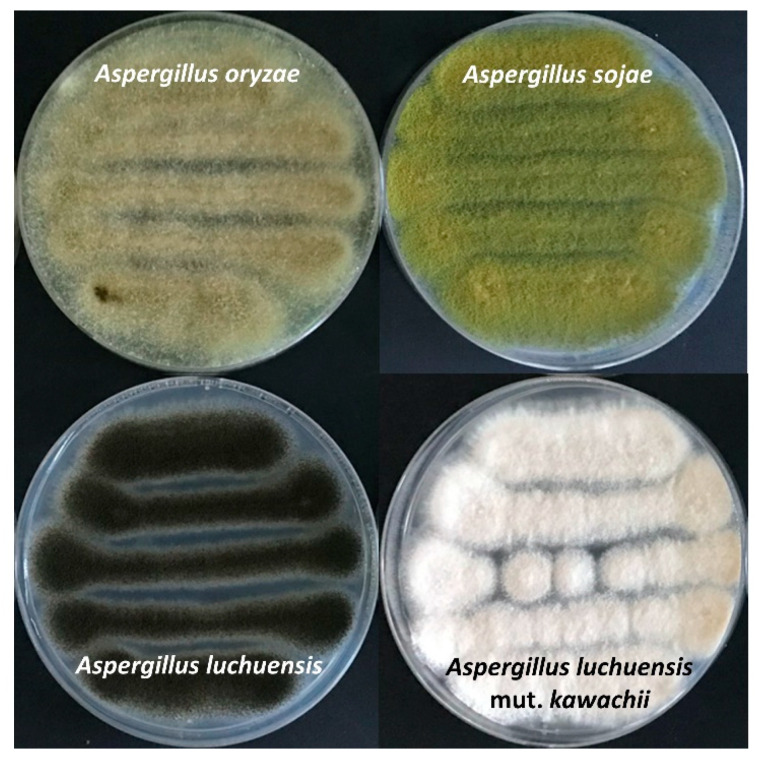
Plate culture images of *A. oryzae*, *A. sojae*, *A. luchuensis* and *A. kawachii*.
